# The Time to Reconsider Mineralocorticoid Receptor Blocking Strategy: Arrival of Nonsteroidal Mineralocorticoid Receptor Blockers

**DOI:** 10.1007/s11906-022-01177-6

**Published:** 2022-04-30

**Authors:** Yuta Tezuka, Sadayoshi Ito

**Affiliations:** 1 Division of Nephrology, Endocrinology and Vascular Medicine, Graduate School of Medicine, Tohoku University, 1-1 Seiryo-cho, Aoba-ku, Sendai, Miyagi 980-8574 Japan; 2 Katta General Hospital, Shiroishi, Miyagi Japan

**Keywords:** Esaxerenone, Finerenone, Nonsteroidal mineralocorticoid receptor blocker, Mineralocorticoid receptor, Hypertension

## Abstract

**Purpose of Review:**

The study aims to verify the advantages of nonsteroidal mineralocorticoid receptor blockers (MRBs) in the management of hypertension and cardiovascular and renal diseases, comparing with conventional MRBs.

**Recent Findings:**

Based on the unique structures, the nonsteroidal MRBs have higher selectivity for mineralocorticoid receptors (MRs) and show no agonist activity for major steroid hormone receptors in contrast to steroidal MRBs. Today, there are two nonsteroidal MRBs, esaxerenone and finerenone, which completed phase 3 clinical trials. Series of clinical trials have shown that both agents achieve similar MR blockade with smaller doses as compared with steroidal MRBs, but have no off-target side effect such as gynecomastia. Esaxerenone has persistent blood pressure-lowering effects in various hypertensive populations, including essential hypertension and those with diabetes and/or chronic kidney disease, while finerenone has demonstrated reduction of the cardiovascular risk rather than blood pressure in patients with diabetes and chronic kidney disease.

**Summary:**

Nonsteroidal MRBs are a more refined agent which contributes to appropriate MR blocking with minimized unpleasant adverse effects.

## Introduction

Compelling evidence highlights that blocking of mineralocorticoid receptors (MRs) could benefit to various hypertensive patients, regardless of the actual levels of peripheral aldosterone [1–3, 4•, 5–8]. Since the discovery in 1957 of a unique class of synthetic steroids, “spirolactones” which abolish aldosterone effects [9], several mineralocorticoid receptor blockers (MRBs) have been developed and proven to contribute to organ protection, particularly in heart failure (HF), diabetic kidney disease (DKD), and chronic kidney disease (CKD), beyond its antihypertensive effects [8, 10–12]. In the current situation, spironolactone (SC-9420) and eplerenone (SC-66110, CGP-30083) are two major steroidal MRBs supported by abundant clinical evidence from many momentous trials, while those studies also elucidate limitations of those steroidal MRBs in hypertension treatment, including both renal-related and off-target side effects [13–15].

For the last 6 decades, continuous efforts to achieve more safe and efficient inhibition of MRs have advanced towards development of a novel class of nonsteroidal MRBs, employing high-throughput screening [14, 16, 17]. Several compounds of nonsteroidal MRBs have proceeded with clinical trials, and in 2019, esaxerenone (CS-3150) was first marketed for hypertension in Japan [18]. Esaxerenone is a novel oral selective nonsteroidal MRB which has a longer half-life and higher selectivity for MRs than traditional MRBs [16, 19]. With the great selectivity for MRs, esaxerenone has demonstrated its solid effects on blood pressure (BP) without side effects related to sex steroid hormone receptors (SSHRs) such as gynecomastia [20, 21, 22••, 23••]. Those studies also reported a significant reduction of urinary albumin excretion in patients with DKD under esaxerenone treatment [23••, 24]. Finerenone (BAY 94–8862), another nonsteroidal MRB, has just been approved by the U.S. Food and Drug Administration (FDA) in July 2021. Several lines of evidence confirmed that finerenone can safely and efficiently reduce the cardiovascular and renal risk in patients with DKD, CKD, and HF [25, 26•, 27••]. Nonsteroidal MRBs are, therefore, in the spotlight as a new key player in hypertension management.

Herein, we review recent findings of nonsteroidal MRBs, particularly esaxerenone and finerenone, in the field of hypertension, their clinical impacts, and potential, comparing with traditional MRBs. In this article, we also argue what we could expect from nonsteroidal MRBs to provide, and remaining issues to be further investigated for our future practice.

## Different Blocking Profiles of Steroid Hormone Receptors by Steroidal and Nonsteroidal MRBs Based on Their Structural Features

**Fig. 1:**
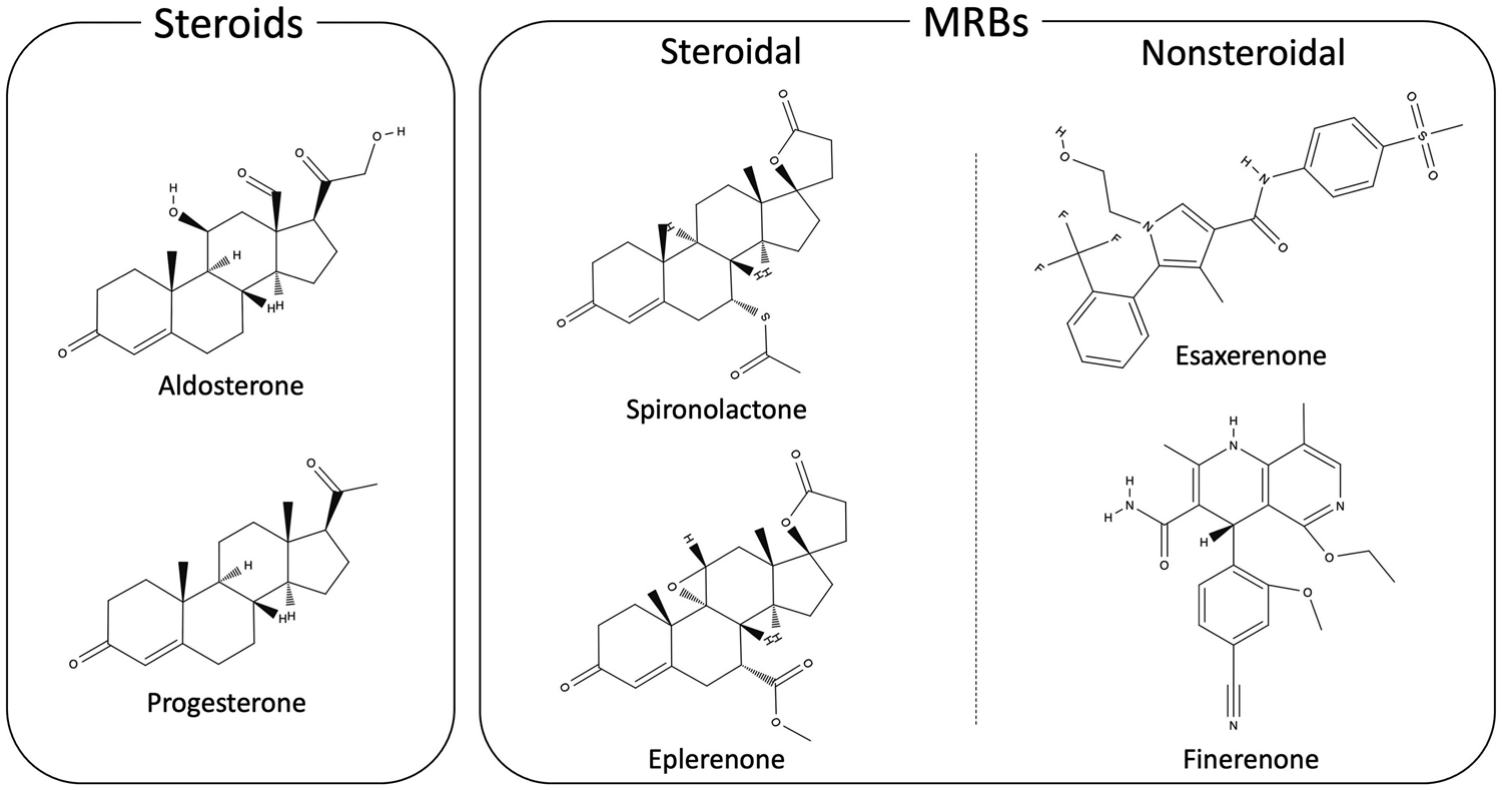
Molecular structures of steroids and mineralocorticoid receptor blockers. The structures of steroidal and nonsteroidal mineralocorticoid receptor blockers (MRBs) are shown, compared with aldosterone, a representative mineralocorticoid in human, and progesterone. Spironolactone was generated by mimicking the structure of progesterone, while eplerenone was developed to improve the selectivity of spironolactone for mineralocorticoid receptors. Nonsteroidal MRBs are relatively “bulky” and quite distinct molecules from those steroidal agents

**Fig. 2:**
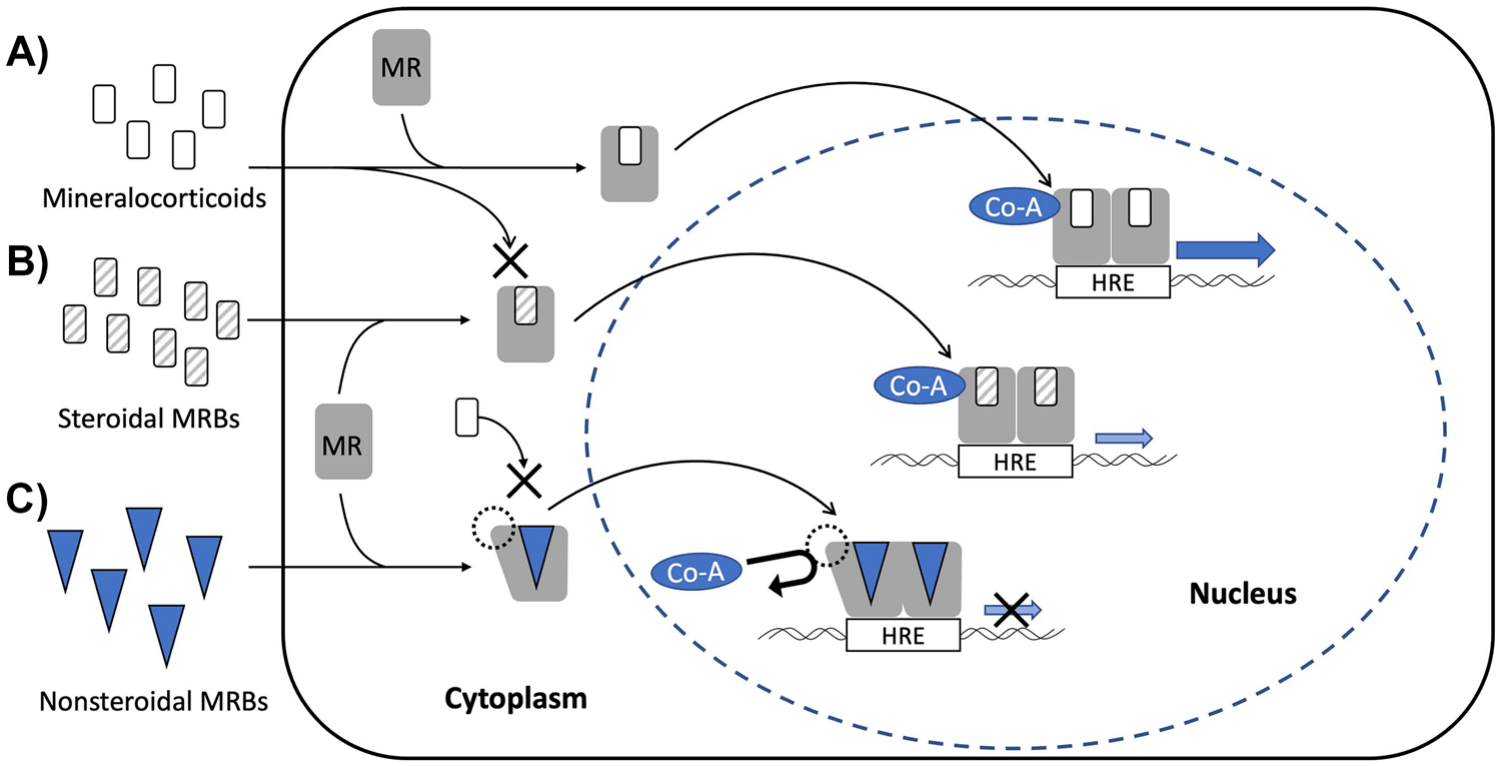
Binding mode of mineralocorticoid receptor blockers to mineralocorticoid receptors. Mineralocorticoids (**A**) and mineralocorticoid receptor blockers (MRBs; **B** and **C**) bind to mineralocorticoid receptors (MRs) in the cytoplasm. (A) The complexes of a mineralocorticoid and a MR form a homodimer after translocation into the nucleus. Then, the homodimer complex binds to the hormone responsive element (HRE) of the target gene, leading to DNA transcription supported by co-activators (Co-A). (B) Steroidal MRBs prevent mineralocorticoids from binding to MRs, but themselves have partial agonist activity for MRs. Therefore, steroidal MRBs could also somewhat prompt transcription of the target gene. (C) Due to the “bulky” structure, nonsteroidal MRBs alter the shape of MR when binding. This modified MR complex precludes not only mineralocorticoid binding but also the recruitment of co-activators, resulting in inhibition of MR signaling

As traditional MRBs originated from synthetic agents with structural elements of progesterone [14], both spironolactone and eplerenone have a steroidal structure which enables them to smoothly pass across cell membrane as mineralocorticoids (Fig. 1). Those drugs inhibit mineralocorticoids from activating MR signaling by competitively binding to MRs in the cytoplasm, resulting in reduction of BP and inflammation (Fig. 2) [28]. Both MRBs also act as a weak agonist for MRs [16]. In contrast, nonsteroidal MRBs show unique mode of MR antagonism. For esaxerenone, the chemical structure is relatively large and quite distinct from those of traditional MRBs (Fig. 1) [16]. Reflecting the difference between their structures, the crystallization experiment demonstrated that esaxerenone shares the same binding site of MRs with other steroidal MRBs, but additionally extended into the binding pocket [29•]. This deep binding of esaxerenone leads to its very high affinity for MRs, compared with spironolactone and eplerenone: IC_50_ values are 3.7, 66, and 970 nM, respectively (Table 1) [16]. Moreover, esaxerenone does not have any agonist potency for human MRs due to the modified MR structure by esaxerenone which precludes coactivators from binding to MRs (Fig. 2) [29•]. Besides, finerenone also has a “bulky” structure with higher potency for MRs (a IC_50_ value of 18 nM) than the steroidal MRBs (Fig. 1) [17]. Intriguingly, finerenone has been reported not only to inhibit aldosterone-MR complex formation but also to decrease basal gene transcription as an inverse antagonist [30]. This reverse effect may result from the protrusion of MR helix 12 due to finerenone binding, which suppresses coactivator recruitment for MR signaling as esaxerenone [30]. From an aspect of pharmacokinetics, esaxerenone has a longer half-life time (18.6 h) than spironolactone and its major metabolites (up to 16.5 h), and eplerenone (up to 6 h), whereas finerenone has the shortest half-life time (around 2 h) among them (Table 1) [16, 31–33]. Despite the less lipophilic nature of the nonsteroidal MRBs than the others, esaxerenone and finerenone enter cells as steroidal MRBs, and exhibit antihypertensive effects, including natriuresis, and antiinflammatory and antifibrotic response in kidneys, heart, and blood vessels, by abrogating MR activation [14, 34, 35].

**Table 1:** Summary of mineralocorticoid receptor blockers

	Spironolactone	Eplerenone	Esaxerenone	Finerenone
Drug code	SC-9420	SC-66110, CGP-30083	CS-3150	BAY 94–8862
Drug type	Steroidal	Steroidal	Nonsteroidal	Nonsteroidal
Half-life time	Up to 16.5 h (including its metabolites)	4–6 h	18.6 h	2–3 h
Affinity (IC_50_ values, nM)				
For MRs	66	970	3.7	18
For GRs	2600	36,000	> 5000	> 10,000
For ARs	640	42,000	> 5000	> 10,000
For PRs	180	7400	> 5000	> 10,000
Agonist activity for MRs	Weak	Weak	None	None
Clinical utility				
Blood pressure	Effective	Effective	Effective	Relatively weak?
Organ protection	Effective	Effective	Potentially effective	Effective
Side effects				
Renal-related	Common	Less frequent	Less frequent	Less frequent
SSHR-related	Common	Less frequent	None	None
Availability	Worldwide	Worldwide	Only in Japan	Approved by FDA

*MR* mineralocorticoid receptor, *GR* glucocorticoid receptor, *AR* androgen receptor, *PR* progesterone receptor, *SSHR* sex steroid hormone receptor, *FDA* food and drug administration

Of note, those nonsteroidal MRBs show different distribution patterns from the steroidal MRBs. Experiments using quantitative autoradiography indicated that both radio-labeled spironolactone and eplerenone show a higher accumulation of drug-equivalent concentrations in kidneys than in cardiac tissues in rodents [36], while radio-labeled esaxerenone and finerenone have balanced accumulation between kidneys and heart [37, 38]. However, what this distributional difference means in the real-world practice remains unknown, although a few animal studies have reported slight differences in cardiac protective effects between nonsteroidal and steroidal MRBs [34, 38]. For central nerve system, it was common across all examined MRBs that the radioactivity in brain was significantly lower than in blood.

In addition, nonsteroidal MRBs show greater selectivity for MRs compared with traditional MRBs. Due to the imitated structure for progesterone, spironolactone and its metabolites possess certain binding ability for various steroid hormone receptors [14]. Evaluation of the transcriptional activity of those steroidal receptors demonstrated that spironolactone blocks ligand binding to human glucocorticoid receptors (GR), androgen receptors (AR), and progesterone receptors (PR) with IC_50_ values of 2600, 640, and 180 nM, respectively (Table 1) [16]. Furthermore, spironolactone also acts as a moderate agonist for ARs and PRs, contributing to off-target side effects such as gynecomastia and irregular menstruation [15]. To compensate for those unfavorable nature of spironolactone, eplerenone was synthesized as a more selective MRB. In particular, IC_50_ values of eplerenone for ARs and PRs are increased to 42,000 and 7400 nM, respectively, while its IC_50_ value for MRs is also increased from 66 to 970 nM (Table 1) [16]. In contrast, esaxerenone and finerenone have very high affinity for MRs and no agonist or antagonist effect on the other steroid hormone receptors as in vitro experiments demonstrated [16, 17]. This very high selectivity for MRs is considered attributable to the side-chain rearrangement by nonsteroidal MRBs which could occur in MRs, but not in GRs, ARs, and PRs [29•]. Accordingly, nonsteroidal MRBs are expected to exert MR-blocking effects with relatively small doses, following less side effects, particularly in SSHR-associated ones.

## Clinical Evidence of Nonsteroidal MRBs in Hypertension

### BP-Lowering Effects

To date, in nonsteroidal MRBs, only esaxerenone has been launched as an antihypertensive agent in Japan. For monotherapy with esaxerenone, a phase 2 double-blind study enrolling 426 patients with essential hypertension (EH) without CKD showed the significant decrease of sitting BP after 12-week treatment compared with the placebo group: the least squares mean changes (95% confidence interval) in systolic and diastolic BP were − 7.0 (− 9.5, − 4.6)/ − 3.8 (− 5.2, − 2.4) mmHg in the placebo group, and − 10.7 (− 13.2, − 8.2)/ − 5.0 (− 6.4, − 3.6), − 14.3 (− 16.8, − 11.9)/ − 7.6 (− 9.1, − 6.2), and − 20.6 (− 23.0, − 18.2)/ − 10.4 (− 11.8, − 9.0) mmHg in the esaxerenone groups of 1.25, 2.5, and 5.0 mg once daily, respectively [20]. The BP-lowering effects were dose-dependent and similar even when esaxerenone was initiated in combination with a calcium channel blocker or an inhibitor of renin-angiotensin system (RAS) [21, 22••]. Of note, the subgroup analysis showed that BP reduction under esaxerenone is numerically greater in females, elderly, and those with lower levels of plasma renin activity [22••]. In comparison between esaxerenone and eplerenone, a phase 3 clinical trial, ESAX-HTN study, including 1001 adult patients with EH, demonstrated that esaxerenone (2.5 mg daily) is not inferior to eplerenone (50 mg daily) for the antihypertensive effect on clinic sitting BP [22••]. In this study, the proportion of the patients who achieved target BP levels (< 140/90 mmHg) were 31.5, 41.2, and 27.5% in the esaxerenone groups (2.5 and 5 mg daily) and the eplerenone group (50 mg daily), respectively [22••]. Ambulatory BP monitoring also showed similar BP trends between 2.5 mg/day of esaxerenone and 50 mg/day of eplerenone within daytime, while nighttime systolic BP was more dramatically improved in the esaxerenone group than the eplerenone group [39].

The antihypertensive effect of esaxerenone was further assessed for the following populations: CKD, DKD, and primary aldosteronism (PA). In hypertensive patients with CKD (estimated glomerular filtration ratio [eGFR] between 30 and 60 mL/min/1.73 m^2^), 5 mg/day of esaxerenone monotherapy significantly lowered BP by − 18.5 (− 23.7, − 13.3)/ − 8.8 (− 11.9, − 5.7) mmHg with no renal-related adverse effect [24]. Similar BP improvement was also observed with smaller doses of esaxerenone (median: 2.5 mg/day) in CKD patients under treatment with a RAS inhibitor [24]. In hypertensive type 2 DKD patients, add-on therapy of esaxerenone to a RAS inhibitor significantly decreased BP by − 13.7 (− 17.6, − 9.8)/ − 6.2 (− 7.8, − 4.6) mmHg, and reduced urine albumin-to-creatinine ratio (UACR) by about 30% [40]. The antihypertensive effects were consistent across subgroups of age and glycemic control. Finally, the efficacy of esaxerenone was evaluated in PA patients. In the study with 44 PA patients, esaxerenone was initiated at 2.5 mg/day and titrated to 5 mg/day [41•]. At the end of 12-week treatment, 93% (41/44) of study participants were treated with 5 mg/day of esaxerenone, and 68% had concomitant antihypertensive agents, a calcium channel blocker or an alpha blocker. Under esaxerenone treatment, BP was decreased by − 17.7 (− 20.6, − 14.7)/ − 9.5 (− 11.7, − 7.3) mmHg, resulting in 47.7% achievement of target BP levels (< 140/90 mmHg) in PA [41•]. Those findings indicate that esaxerenone possesses at least equivalent BP-lowering ability to other steroidal MRBs in various conditions, although all the clinical data shown here were obtained from Japanese cohorts.

In contrast, international trials targeting for chronic HF and CKD suggested that finerenone has a relatively weak effect on BP compared to esaxerenone. In the ARTS (MinerAlocorticoid Receptor antagonist Tolerability Study) trial, the reductions of systolic BP were significantly smaller in the finerenone group (the dose of 10 mg/day: mean ± standard deviation, − 4.2 ± 15.5 mmHg) than in the spironolactone group (the mean dose of 37 mg/day: − 10.1 ± 15.0 mmHg) [25]. Similarly, the decrease of systolic BP by finerenone was relatively small, but similar to eplerenone in the ARTS-HF (MinerAlocorticoid Receptor antagonist Tolerability Study- Heart Failure) trial: the least squares mean changes of systolic BP were − 2.365 (− 5.287, 0.558) mmHg in the eplerenone group (the average dose of 38.6 mg/day) and − 0.825 (− 3.929, 2.278), − 2.532 (− 5.630, 0.566), − 2.697 (− 5.757, 0.363), and − 2.397 (− 5.348, 0.554) mmHg in the finerenone groups (2.5 to 5, 5 to 10, 7.5 to 15, and 10 to 20 mg daily, respectively) [42]. Those results were consistent with those of its phase 3 trial, FIDELIO-DKD (Finerenone in Reducing Kidney Failure and Disease Progression in Diabetic Kidney Disease) [27••]. However, those studies were designed to evaluate the benefits of finerenone treatment on cardiac and renal outcomes, but not on BP. Due to the limited analysis on BP, the actual antihypertensive effect of finerenone should be further investigated.

### Protective Effects on Cardiac and Renal Diseases

Several Japanese studies indicate the antialbuminuric effect of esaxerenone in DKD. In a study enrolling 51 patients with type 2 diabetes and albuminuria under a RAS inhibitor, esaxerenone initiation improved not only BP but also UACR during a 12-week observational period [40]. In this study, the dosage of esaxerenone was gradually titrated from 1.25 to 5 mg/day based on monitoring of serum potassium and eGFR, and at the end, 15.7, 47.1, and 37.3% of patients were treated with 1.25, 2.5, and 5 mg daily of esaxerenone. Overall, UACR decreased by 32.4% from baseline levels with improvement of urinary β2-microglobulin excretion [40]. Consequently, the ESAX-DN study, a more focused study on microalbuminuria, also demonstrated the long-term effect of esaxerenone on UACR in diabetes patients treated with a RAS inhibitor [23••]. Total 455 diabetes patients with microalbuminuria (UACR within 45 to 300 mg/g creatinine) were randomly allocated to the placebo and the esaxerenone groups, and in the latter group, esaxerenone was initiated at 1.25 mg/day and titrated to 2.5 mg/day. After 52-week treatment with esaxerenone, the UACR was significantly reduced by 58% from baseline values compared with the placebo group (8%) [23••]. Further analysis revealed that this antialbuminuric effect was independent of its BP-lowering effect. Moreover, of these, 22% of patients treated with esaxerenone achieved remission in UACR, which was more frequent than those with placebo (4%) [23••]. Similar improvement of UACR was also observed in a small study of 52 type 2 diabetes patients with macroalbuminuria (UACR > 300 mg/g creatinine): mean UACR decreased to less than half of baseline, and 52% of the study cases no longer had macroalbuminuria after esaxerenone treatment for 28 weeks [43]. From a cardio-protective aspect, a retrospective analysis reported the significant decrease of plasma B-type natriuretic peptide (BNP) levels by esaxerenone treatment in patients with HF [44]. Thus, clinical evidence on organ-protective benefits of esaxerenone is accumulating, but still limited. Future studies on hard outcomes such as CKD progression and the onset of cardiovascular disease are in great demand.

On the other hand, there are two large, randomized trials, ARTS and FIDELIO-DKD, which confirmed improved clinical outcome by finerenone treatment in HF with reduced ejection fraction (HFrEF) and/or DKD [25, 27••]. First, the ARTS study was initially conducted to assess the safety and tolerability of finerenone in patients with HFrEF and DKD, incorporating with 10 countries worldwide. Within the original study, finerenone treatment was associated with reductions of BNP, N-terminal pro-BNP, and UACR as was spironolactone treatment [25]. Furthermore, targeting for a composite outcome of any death, hospitalization for CVD, and emergency presentation for exacerbated HF, those who received once-daily administration of finerenone (10 mg once daily or more) had a lower incidence of the composite endpoint at day 90 than those treated with eplerenone (the average dose of 38.6 mg/day) in the ARTS-HF study [42]. Especially, comparing with the eplerenone group, the hazard ratio (HR) in the 10- to 20-mg group of finerenone was 0.56 (0.35, 0.90) at the composite outcome: HRs, 0.13 (0.02, 1.07), 0.56 (0.34, 0.93), and 0.58 (0.33, 1.02) for any death, cardiovascular hospitalization, and emergency presentation for worsening chronic HF, respectively [42]. In addition, the ARTS-DN study confirmed the dose-dependent improvement of UACR after finerenone initiation [45]. However, we must consider that the ARTS trial was not designed for comparison between finerenone and steroidal MRBs.

Recently, results of the FIDELIO-DKD trial have been announced [27••], advancing finerenone towards FDA approval. This phase 3 trial recruited adults with type 2 diabetes mellitus and CKD treated with a RAS inhibitor across the world, and finally, 5734 patients were randomly allocated to the finerenone or placebo groups. With a median follow-up period of 2.6 years, the finerenone group showed a lower incidence of the renal composite outcome, including kidney failure, over 40% decrease of eGFR and death from renal causes, than the placebo group (17.8% vs. 21.1%, respectively: HR, 0.82 [0.73, 0.93]) [27••]. Particularly, the sustained decrease of eGFR from baseline was less frequent in the finerenone group than in the placebo group (HR, 0.82 [0.72, 0.92]), while neither kidney failure nor death from renal causes was significantly different between them [27••]. For the cardiovascular arm of the trial, the finerenone group also had a lower incidence of the secondary composite outcome of death from CVD, nonfatal CVD, and hospitalization for HF compared with the placebo group (13.0% vs. 14.8%, respectively: HR, 0.86 [0.75, 0.99]) irrespective of a history of CVD [27••, 46]. In addition, subgroup analysis revealed that the incidence of new-onset atrial fibrillation or flutter is lower in the finerenone group than in the placebo group (3.2% vs. 4.5%, respectively: HR, 0.71 [0.53, 0.94]) [26•]. Those outcomes in the finerenone group were obtained with minimal changes of systolic BP from baseline: − 3.0 and − 2.1 mmHg at months 1 and 12 after finerenone initiation, respectively [27••]. Finerenone is, therefore, expected as an organ-protective agent rather than an antihypertensive one in type 2 diabetes.

## Safety Profiles of Nonsteroidal MRBs: Renal-Related and Off-target Side Effects

Basically, decrease of eGFR followed by elevation of serum potassium is closely tied to the MR-blocking effect under MRB treatment. In patients with essential hypertension and preserved renal function (eGFR > 60 mL/min/1.73 m^2^), esaxerenone monotherapy lowered eGFR depending on its doses: the mean eGFR changes with standard deviation between baseline and week 12 were − 2.31 ± 6.85, − 3.69 ± 7.98, and − 6.36 ± 8.08 mL/min/1.73 m^2^ in the 1.25-, 2.5-, and 5-mg daily groups, respectively, while those of placebo and eplerenone (50 to 100 mg daily) were 0.06 ± 6.05 and − 2.11 ± 6.35 mL/min/1.73 m^2^, respectively [20]. Concurrently, serum potassium levels elevated under esaxerenone treatment by 0.2 to 0.3 mM at weeks 1 and 2, and then gradually decreased to nearly baseline levels. Finally, in the phase 2 trial, adverse events of hyperkalemia or renal dysfunction were reported in 0, 3.6, and 3.4% and 3.6, 0, and 3.4% of the esaxerenone groups of 1.25, 2.5, and 5 mg daily, respectively, compared with 2.3 and 1.1% of the placebo group [20]. Consequently, the phase 3 trial also confirmed that the incidence of renal-related adverse effects was not different between treatment of eplerenone and esaxerenone [22••]. With careful assessment of those parameters, esaxerenone could be safely used in patients with moderate renal dysfunction (eGFR between 30 and 60 mL/min/1.73 m^2^) and those who treated with a RAS inhibitor [24, 40], while when used in combination with a RAS inhibitor, esaxerenone use tended to more frequently cause serum potassium elevation (12.1%) and eGFR decrease (5.2%) [24].

Probably reflecting the mild effect on BP, finerenone in combination with other antihypertensives was associated with a lower incidence of hyperkalemia and worsening renal function than spironolactone in the patients with HFrEF and CKD: 4.5 and 10.4% in the 10-mg daily finerenone group, and 11.1% and 38.1% in the 25- to 50-mg daily spironolactone group, respectively [25]. On the other hand, in the ARTS-HF trial, the incidence of hyperkalemia (> 5.5 mM) and the degree of eGFR decline were similar between eplerenone and finerenone groups [42]. The prevalence of those adverse events by finerenone was compatible with the results of FIDELIO-DKD trial [27••]. Accordingly, renal-related side effects by nonsteroidal MRBs are considered almost equivalent to those of eplerenone, and maybe less frequent than those of spironolactone.

During trials of esaxerenone and finerenone, nonspecific symptoms, including nasopharyngitis, upper respiratory tract inflammation, and headache, were commonly observed across the study groups of the placebo, nonsteroidal MRBs, and other steroidal MRBs [20, 22••, 25, 42]. Expectedly and importantly, no adverse effect related to SSHRs was reported in the patients treated with nonsteroidal MRBs. In addition, there were no other significant differences of the safety profiles between nonsteroidal and steroidal MRBs. Liver function plays a key role for the metabolism of nonsteroidal MRBs as cytochrome P450 3A4 predominantly regulates its metabolic process. Nevertheless, evaluation of their pharmacokinetics suggested that those nonsteroidal MRBs could be safely used in mild to moderate hepatic impairment [47, 48].

## Perspectives of MR Blocking Strategy

Recent studies have provided compelling evidence that aberrant MR activation is deeply involved in end-organ damage. Several factors such as sympathetic hyperactivity and obesity cause MR overactivation via aldosterone excess, leading to pro-inflammatory immune responses and vascular fibrosis [49, 50]. Without aldosterone, salt intake also induces Rac1-mediated MR activation in the target tissues, heart, and kidneys [51–53]. As another mechanism, glucocorticoids could act as an agonist for MRs in the condition where inactivation of cortisol by 11β-hydroxysteroid dehydrogenase type 2 is impaired by endogenous and exogenous factors [54]. Therefore, in hypertensive cases, MRBs could be initiated to abolish such MR-related organ damage regardless of aldosterone levels.

While accumulating evidence has established the solid position of spironolactone as an add-on drug in patients with resistant hypertension and CVD, SSHR-related adverse events often preclude them from continuing spironolactone. In the RALES (Randomized Aldactone Evaluation Study) trial enrolling severe HF patients, spironolactone use was associated with a higher risk for gynecomastia or breast pain in men compared with the placebo (10% vs. 1%, respectively) [55]. Similarly, such adverse events were reported in 10% of men treated with spironolactone for resistant hypertension [56]. In women, irregular menstruation is reported as the most common adverse effect of spironolactone [57]. In contrast, with decreased affinity for SSHRs, eplerenone less frequently cause those symptoms, but does not completely resolve them [58]. Also, due to the shorter half-life time and the lower potency compared with spironolactone, eplerenone needs to be taken twice daily [59], possibly leading to poor adherence.

As forementioned, the notable advantage of nonsteroidal MRBs is their outstanding selectivity for MRs (Table 1). Based on the unique binding mode, both esaxerenone and finerenone have no agonist activity for MRs and cause no sexual adverse effects. Furthermore, the results of several trials indicate that once daily administration of those MRBs leads to antihypertensive and organ-protective benefits at least equivalent to steroidal MRBs. Particularly, esaxerenone is considered useful as a standard antihypertensive drug in everyday practice. Therefore, the nonsteroidal agents enable us to extend MR-blocking therapy even to the patients who cannot pursue the treatment with conventional MRBs because of sexual side effects and/or poor adherence, resulting in better clinical outcomes of CVD. Besides, it also seems worthy investigating additional benefits specific to nonsteroidal MRBs, e.g., action on arteriosclerosis and cerebrovascular disease. Inversely, spironolactone may still serve as a key drug in certain endocrine-related conditions involving overactivation of other steroid hormone receptors. Recent studies focusing on steroid profiles revealed disorganized production of multiple steroid hormones in endocrine disorders [60–62]. However, a common thing in both steroidal and nonsteroidal MRBs is that their effects are closely associated with renal function and serum potassium. We further need to determine proper selection of and management with MRBs depending on the patient’s condition.

## Conclusion

As expected, nonsteroidal MRBs, esaxerenone and finerenone, have great potential in management of hypertension and its complications. Their distinctive structures allow themselves to rigidly bind to just MRs, but not other steroid hormone receptors. Consequently, with no off-target sexual symptoms, the intended effect of MR blocking can be efficiently achieved by initiation of nonsteroidal MRBs. Recent trials confirmed the antihypertensive effect of esaxerenone in both monotherapy and combination therapy, and the preventive effects of nonsteroidal MRBs on cardiac and renal damage. In addition, other candidates of nonsteroidal MRBs are being examined in rapid succession [63–65]. Although further examination on their clinical utility is required to enrich our understanding about those agents, nonsteroidal MRBs will contribute to lowered risks for CVD and CKD progression, particularly in the patients who are intolerant of steroidal MRBs. A new era is coming to reappraise our antihypertensive strategy using MRBs.
